# Transfusion-related acute lung injury (TRALI) following intravenous immunoglobulin infusion in a rituximab immunosuppressed patient with long-shedding SARS-CoV-2

**DOI:** 10.1186/s12879-024-09809-9

**Published:** 2024-09-04

**Authors:** Ganna Degtiarova, Anna Conen, Alexander Klarer, Teuta Arifi, Gina Guldimann, Sebastian Finkener, Andres Spirig, Hans-Joachim Kabitz

**Affiliations:** 1 Department of Internal medicine, Medical University Clinic, Kantonsspital Aarau, Aarau, Switzerland; 2 Clinic for Infectious Diseases and Infection Prevention, Medical University Clinic, Kantonsspital Aarau, Aarau, Switzerland; 3 Clinic for Intensive Care Medicine, Kantonsspital Aarau, Aarau, Switzerland; 4 Department of Neurology, Medical University Clinic, Kantonsspital Aarau, Aarau, Switzerland; 5 Institute of Radiology, Kantonsspital Aarau, Aarau, Switzerland; 6 Department of Pulmonary and Sleep Medicine, Medical University Clinic, Kantonsspital Aarau, Aarau, Switzerland

**Keywords:** Acute lung injury, Transfusion reaction, Human intravenous immunoglobulin, SARS-CoV-2, IgG Deficiency, Immunosuppression

## Abstract

**Background:**

Transfusion-related acute lung injury (TRALI) is a rare life-threatening complication of blood product transfusion. Intravenous immunoglobulin (IVIG)-related TRALI is scarcely reported.

**Case presentation:**

A 63-year-old male patient suffering from multiple sclerosis treated with half-yearly rituximab infusions, was hospitalized due to dry cough, daily fever and shivering for seven days despite antibiotic therapy. Because of the history of COVID-19 one month prior without the symptoms having improved since, persistent bilateral multifocal areas of ground glass opacities in chest computed tomography and positive SARS-CoV-2 PCR from bronchoalveolar lavage with a cycling time of 30.1 COVID-19 due to long-shedding SARS-CoV-2 under immunosuppression with rituximab was diagnosed. He received treatment with nirmatrelvir und ritonavir and because of diagnosed IgG deficiency additionally a single dose of 20 g IVIG. During the IVIG infusion, the patient acutely developed tachycardia, hypotension, fever, chills, and hypoxemic respiratory failure due to pulmonary edema. TRALI was promptly diagnosed, and the patient was transferred to the intensive care unit for non-invasive ventilation for less than 24 h. The patient was discharged home from regular ward 72 h later in a good general condition and no remaining symptoms of TRALI.

**Conclusion:**

IVIG-related TRALI is a rare but life-threating condition and prompt recognition is lifesaving. Due to an increased use of IVIG not only in long-shedding SARS-CoV-2, an increase of TRALI incidence is expected.

## Introduction

Transfusion-related acute lung injury (TRALI) is a rare and life-threatening complication of blood product transfusion resulting in acute respiratory distress and non-cardiogenic pulmonary edema [1]. A systemic inflammatory process with damage to the alveolar-capillary barrier of the lung by neutrophil activation has been suggested as the main pathophysiological mechanism of TRALI [2]. The accurate assessment of the incidence of TRALI is difficult due to poor recognition, passive reporting, and several redefinitions of TRALI due to a better understanding of the pathophysiology of this complication. The reported incidence of TRALI ranges between 0.08% and 1.12% per administered product and is primarily related to blood transfusion, with only 17 cases of TRALI related to the administration of intravenous immunoglobulin (IVIG) reported between 1990 and 2019 [3, 4]. With the increasing use of IVIG for the treatment of a wide range of diseases, the total numbers of TRALI are rising, underlining the importance of prompt recognition, as immediate treatment is lifesaving.

We report a clinical case of TRALI following IVIG administration in a patient immunosuppressed with rituximab due to multiple sclerosis suffering from long-shedding SARS-CoV-2.

## Case presentation

We report on a 63-year-old male patient who was admitted to our tertiary care hospital (Kantonsspital Aarau, Switzerland) because of a deterioration of the general condition with daily fever and shivering for seven days, as well as a dry cough, despite antibiotic therapy prescribed by the general practitioner for a suspected pulmonary infection. One month prior, the patient suffered from COVID-19 and received an outpatient treatment with nirmatrelvir und ritonavir for five days. Since then, he felt persistently tired and coughed. His medical history included a relapsing-remitting multiple sclerosis (MS), which was diagnosed twenty-two years ago and has been treated with half-yearly infusions of rituximab in recent years. The last infusion of rituximab was 4 months ago. In addition, he had been treated for diffuse large B-cell lymphoma six years prior and has been in complete clinical and radiological remission. He has never suffered from heart disease and his last echocardiography three months before admission was normal. The patient has been vaccinated three times against COVID-19 (Pfizer/BioNTech), the last vaccination being 1.5 years ago.

**Fig. 1:**
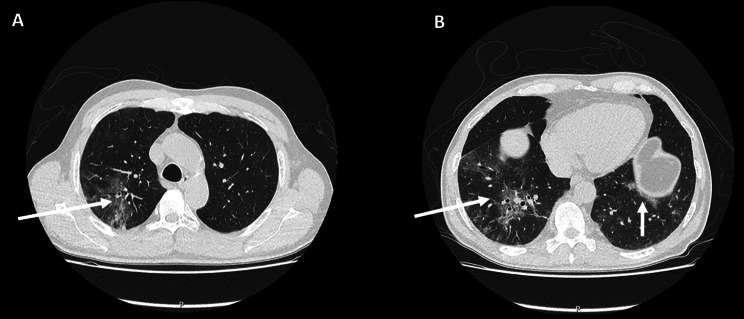
Chest computed tomography acquired on the admission day (3 days before IVIG Infusion). (**A**) Multifocal ground glass opacities in the right upper lobe (white arrow. (**B**) Multifocal ground glass opacities in the in the right and left lower lobe (white arrows)

**Fig. 2:**
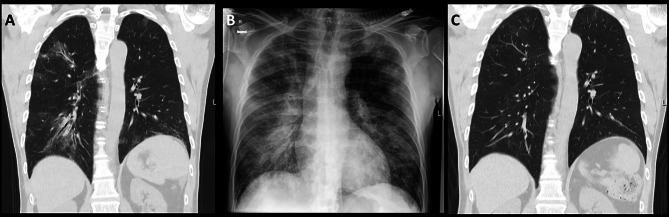
Chest computed tomography acquired on the admission day (three days before IVIG Infusion) with multifocal patchy pulmonary ground glass opacities, accentuated on the right side (**A**). Chest X-Ray acquired during acute respiratory deterioration (**B**) with progressive bilateral opacifications, consistent with non-cardiac pulmonary edema. Chest computed tomography acquired 2 months after discharge from the hospital with almost complete regression of the ground-glass opacities (**C**)

Laboratory examinations at admission showed elevated C-reactive protein of 59 mg/l (normal range < 5 mg/l) and lactate dehydrogenase of 329 IU/l (normal range 120–250 IU/l) in addition to an IgG deficiency with 3.59 g/l (normal range 7–16 g/l). A chest computed tomography revealed bilateral multifocal areas of ground glass opacities, dominant in the right upper and lower lobes (Fig. 1, 2 A). A diagnostic bronchoscopy was performed and microbiological examination of bronchoalveolar lavage fluid showed no bacteria or fungi. The respiratory multiplex PCR only detected SARS-CoV-2 with a cycling time (ct) of 30.1. With the history of COVID-19 one month prior without the symptoms having improved since, these findings were interpreted in the context of COVID-19 due to long-shedding SARS-CoV-2 under immunosuppression with rituximab. Treatment included an extended oral antiviral therapy with nirmatrelvir und ritonavir for ten days. Additionally, due to the impaired humoral immunity secondary to the rituximab therapy and the documented IgG deficiency, the patient received a single dose of 20 g (0.3 g/kg body weight, 200 ml) of IVIG (Privigen^®^, CSL Behring AG, Switzerland). Treatment also included dalteparin for thromboprophylaxis during hospitalisation and the patient’s regular medication, magnesium per os and inhaled salmeterol/fluticason for polyallergenic bronchial asthma, which was changed to inhaled ipratropium bromide/salbutamol during hospitalization. During the transfusion of IVIG there was a sudden onset of shivering, fever up to 40.6° Celsius and tachycardia with 158 beats/min. Initial blood pressure was 114/66 mmHg. At this point, despite the nurses’ warnings, the patient ignored the symptoms, attributed them to pre-existing conditions, and insisted on continuing the transfusion. Shortly thereafter, he developed hypotension with 99/61 mmHg and hypoxemic respiratory failure with a rapid decline in oxygen saturation despite respiration with a mask reservoir of 14 l of oxygen per minute. IVIG transfusion was immediately stopped. Arterial blood gas analysis showed respiratory alkalosis with partial metabolic compensation (pH 7.46, Pa0_2_ 96 mmHg, PaCO_2_ 27 mmHg, HCO3^−^ 19 mmol/l, lactate 1.7 mmol/l). Clinically and radiologically (sonography and chest X-ray), there was evidence of a pulmonary oedema (Fig. 2B). Electrocardiogram (ECG) was normal without signs for ischemia. The N-terminal prohormone of brain natriuretic peptide (NT-proBNP) was increased to 2043 ng/l (normal range < 165 ng/l). The patient received one-time 20 mg intravenous furosemide treatment and was transferred to the intensive care unit (ICU) where non-invasive ventilation (FiO2 65%, PEEP 6 mbar, p_support_ 3 mbar) was started. There was a rapid clinical improvement and after two hours the patient was de-escalated to nasal high-flow therapy (40 l/min/FiO2 40%) and after three hours to short-term nasal cannula oxygenation, allowing transfer back to the normal ward with adequate oxygenation without additional oxygen supplementation less than 24 h later. Also, the fever subsided quickly. The transient leukocytosis during TRALI, with a leukocyte elevation to a maximum of 14.66 G/L, quickly returned to the normal range of 6.9 G/L (normal range 4–10 G/L). The signs and symptoms were interpreted as TRALI Type I following IVIG administration. Three days later the patient was discharged home in a significantly improved general condition and no measurable remaining impairments from TRALI. Antiviral treatment with nirmatrelvir und ritonavir was continued for totally ten days. Two months later the patient is without any clinical symptoms and with significant regression of radiological signs of COVID-19 (Fig. 2C).

## Discussion

TRALI is a leading cause of transfusion-related mortality. The latest revision of the TRALI criteria proposed in 2019 includes acute onset of symptoms within six hours of transfusion, including hypoxemia (PaO2/FiO2 ≤ 300 mmHg or spO2 < 90% at room air), bilateral pulmonary edema on imaging and no evidence of left atrial hypertension – all of which were present in our patient [5]. The updated TRALI definition divides TRALI into two types according to the presence or absence of acute respiratory distress syndrome (ARDS) risk factors. Notably, only after the redefinition in 2019 can TRALI be diagnosed rather than determined by exclusion criteria, as the presence of ARDS risk factors or ARDS no longer doubts the diagnosis of TRALI, but only defines its type. Considering that our patient did not have any risk factors for ARDS, such as aspiration, toxic inhalation, shock, sepsis, pneumonia, multiple trauma, burns, acute pancreatitis, cardiopulmonary disease, and that his COVID19 pneumonitis was stable without respiratory deterioration during the last month, we have classified the clinical case as TRALI type I.

Whereas most reports on TRALI are related to the transfusion of whole blood, red blood cells, platelet concentrates, and transfusion of fresh-frozen plasma, our clinical case shows an IVIG-related TRALI, which is rarely reported [1, 6]. Patients with hematological malignancies undergoing chemotherapy, heart disease, major surgery, and trauma have been identified as risk groups for developing TRALI [1, 6]. Of note, our patient had none of the above risk factors. The B-cell lymphoma was in complete remission for six years. TRALI after the administration of IVIG for neurological disorders, hypogammaglobulinemia, autoimmune diseases, hematological disorders and Kawasaki`s disease, has been reported in single cases [4, 7]. To the best of our knowledge, this is the first case report of TRALI following the administration of IVIG in a rituximab-treated patient suffering from long-shedding SARS-CoV-2.

Rituximab reduces the function of B-cells by targeting CD20 antibodies on their surface, thereby disrupting antibody production and inhibiting viral clearance in COVID-19 patients. In addition, rituximab-treated patients showed a reduced response to SARS-CoV2 vaccines [8]. This explains the prolonged shedding of SARS-CoV-2 virus and the difficulty in eradicating SARS-CoV-2 in rituximab-immunosuppressed patients. In our patient, a positive COVID-19 test with a high ct value of 30.1 corresponded to a low level of virus, which is unlikely to cause infection in a patient with a non-comprimised immune system, but favoured persistence of the virus in our immunocompromised patient. Taking into account the lack of spontaneous clearance of SARS-CoV-2 during the last month, the prolonged COVID-19 symptoms and the presence of secondary antibody deficiency in our patient, which further impairs humoral immunity, we decided to start intravenous IVIG replacement therapy to support the immune system and avoid an unfavourable prognosis. The infusion of IVIG, like the transfusion of any blood product, may be associated with a transfusion reaction, making a risk-benefit assessment of paramount importance. Despite the development of TRALI, which fortunately could be properly treated, we observed a dramatic clinical and radiological improvement in the course of the disease. We are inclined to believe that IVIG can be considered for the treatment of patients with IgG deficiency and COVID-19, but a careful review of the patient’s clinical history and risk factors should be performed.

There are several other causes of acute respiratory distress that should be differentiated from TRALI, and the distinction between transfusion-associated circulatory overload (TACO) and TRALI may be particularly difficult because of the similarity in clinical presentation. However, no history of cardiac disease, a normal ECG, normal echocardiography (left ventricular ejection fraction 54%, no valve pathologies) three months prior, normal renal function, small volume of fluid transfused (200 ml IVIG and no pretransfusion fluid infusion) and slow IVIG administration argue against TACO. The elevation of NT-proBNP is more typical of TACO, but a significantly elevated NT-proBNP level has also been described in TRALI cases [9], so its diagnostic value in differentiating between these two syndromes is limited. We are inclined to think that an increased NT-proBNP in blood at the time of the TRALI is rather the sign of cardiac stress caused by the release of NT-proBNP from the right heart, stimulated by the pulmonary edema and the critical illness itself, that is why the was no further control of the NT-proBNP performed. In line with this there was a stress-mediated temporal increase in the number of circulating leukocytes during TRALI. On the other hand, we observed the typical symptoms of TRALI: fever, chills and hypotension. Although TRALI is less sensitive to diuretics, in the emergency situation with clinical signs of acute pulmonary edema requiring immediate treatment, only a single 20 mg intravenous dose of furosemide was administered by the physician. It is highly unlikely that the patient’s rapid recovery was due to a single injection of low-dose furosemide.

In general, a 2-hit model has been proposed as the basis of TRALI pathophysiology and both hits are required for the development of TRALI [1, 10, 11], The first hit is represented by the pre-existing clinical condition and predisposition of the recipient (patient risk factors), whereas the second hit is caused by the transfused blood product itself. This first hit promotes a pro-inflammatory environment that activates pulmonary endothelium, neutrophils and promotes their immigration into the lungs. The second hit can be either antibody-mediated (anti-leukocyte antibodies in the transfusion product) or driven by non-antibody factors represented by proteins, lipids, extracellular vesicles and aged blood cells. Both antibody and non-antibody factors target the pulmonary endothelium directly or indirectly through various cells, including neutrophils, monocytes and macrophages, causing TRALI.

As IVIG are produced by a large pool of plasma immunoglobulins, which are obtained from different donors, it is impossible to perform a specific antigen and antibody screening in the recipient and donor, respectively. As TRALI is a clinical diagnosis, antibody detection in the blood is not required, is often negative and then results might even be misleading (and are not available in time in this acute life-threatening condition) [4, 12].

The disease usually resolves within 72 h, but the mortality rate of TRALI remains high and can reach up to 10% [3, 13]. There is no specific therapy available and supportive care is the only cornerstone of managing TRALI. Despite pulmonary edema, routine use of diuretics may be harmful because of the increased risk of severe hypotension [14]. Glucocorticoid administration is controversial and the literature is currently insufficient to support its use [14]. According to the literature, invasive mechanical ventilation is required in more than half of the patients [4]. Our patient did not need invasive mechanical ventilation, he could be discharged from the ICU in less than 24 h and from the hospital in 72 h, which is supposed to be at least partially attributed to the prompt recognition and treatment of TRALI.

The transfusion reaction was reported to a hospital haemovigilance unit and also to the company from which IVIG was purchased. We would like to emphasise the importance of informing the relevant authorities about the transfusion reaction in order to ensure patient safety and minimise morbidity and mortality associated with blood transfusion, as well as to investigate new/evolving trends in transfusion reactions.

## Conclusion

With the increased use of IVIG in all areas of medicine, increased awareness of related complications such as TRALI is of paramount importance. It is remarkable that despite the serious complication, our patient improved rapidly, most likely because TRALI was recognized and treated promptly. Although the administration of IVIG can be lifesaving, its application potentially exposes patients to severe complications which should always be kept in mind by the clinical care team.

## Data Availability

Data are not publicly available due to ethical reasons but can be provided by request.

## Abbreviations

ICU: Intensive care unit
IVIG: Intravenous immunoglobulin
TRALI: Transfusion-related acute lung injury
MS: Multiple sclerosis
ct: Cycling time
